# Assessment of Potential Location of High Arsenic Contamination Using Fuzzy Overlay and Spatial Anisotropy Approach in Iron Mine Surrounding Area

**DOI:** 10.1155/2014/905362

**Published:** 2014-07-07

**Authors:** Thanes Weerasiri, Wanpen Wirojanagud, Thares Srisatit

**Affiliations:** ^1^Department of Environmental Engineering, Faculty of Engineering, Khon Kaen University, Khon Kaen 40002, Thailand; ^2^Centre of Excellence on Hazardous Substance Management, Bangkok 10330, Thailand; ^3^Research Center for Environmental and Hazardous Substance Management, Khon Kaen University, Khon Kaen 40002, Thailand; ^4^Department of Environmental Engineering, Faculty of Engineering, Chulalongkorn University, Bangkok 10330, Thailand

## Abstract

Fuzzy overlay approach on three raster maps including land slope, soil type, and distance to stream can be used to identify the most potential locations of high arsenic contamination in soils. Verification of high arsenic contamination was made by collection samples and analysis of arsenic content and interpolation surface by spatial anisotropic method. A total of 51 soil samples were collected at the potential contaminated location clarified by fuzzy overlay approach. At each location, soil samples were taken at the depth of 0.00-1.00 m from the surface ground level. Interpolation surface of the analysed arsenic content using spatial anisotropic would verify the potential arsenic contamination location obtained from fuzzy overlay outputs. Both outputs of the spatial surface anisotropic and the fuzzy overlay mapping were significantly spatially conformed. Three contaminated areas with arsenic concentrations of 7.19 ± 2.86, 6.60 ± 3.04, and 4.90 ± 2.67 mg/kg exceeded the arsenic content of 3.9 mg/kg, the maximum concentration level (MCL) for agricultural soils as designated by Office of National Environment Board of Thailand. It is concluded that fuzzy overlay mapping could be employed for identification of potential contamination area with the verification by surface anisotropic approach including intensive sampling and analysis of the substances of interest.

## 1. Introduction 

Arsenic contamination to the environment can cause an adverse environmental problem that further impacts health. Exposure to arsenic can result in a variety of human health problems, including various forms of cancer (e.g. skin, lung, and bladder), cardiovascular and peripheral vascular disease, and diabetes. Humans may be exposed to arsenic through inhalation, dermal absorption, and ingestion of food, water, and soil [1, 2]. Arsenic is a naturally occurring element present in both inorganic and organic forms in different environmental and biological samples and its concentrations may be increased by anthropogenic contamination [3]. The major sources of arsenic pollution may be natural process such as dissolution of arsenic containing bedrock/minerals and anthropogenic activities, for example, percolation of water from mines, wood preservatives, agricultural chemicals, and discharge from an uncontrolled mining and metallurgical industry. It is estimated that about 60% of arsenic present in the environment is of anthropogenic origin [4–10].

Regarding arsenic occurrence in nature, it geologically occurs in soil. Besides gold mine activities, arsenic-bearing hydrothermal minerals frequently occur in ores containing copper, iron, tin, nickel, lead, uranium, zinc, cobalt, or platinum [11]. Mine drainage refers to surface water or groundwater becoming contaminated with heavy metals, arsenic, and/or sulfuric acid as the water infiltrates into the mine shafts, pits, coal piles, ores processing structures, and wastes impoundments, such as mine tailings piles and disposal ponds [12, 13]. On January 22, 2001, USEPA published a final arsenic rule in the Federal Register that revised MCL for arsenic from 0.05 mg/L to 0.01 mg/L (10 *μ*g/L) for drinking water [14]. Office of National Environment Board of Thailand (NEB) specified MCL of arsenic in soils as 3.9 mg/kg and 27 mg/kg for agriculture and other usages, respectively [15].

In Thailand there is some evidence of arsenic contamination in the area of Wangsaphung district, Loei province. The line governmental agencies had investigated both surface water and groundwater, revealing that arsenic concentration was less than the MCL of 0.01 mg/L specified by USEPA. However, there are still some findings of sick peoples due to arsenic exposure even though the iron mine had been already closed since 2005. With this suspicion, more extensive investigation in the whole area and in environmental medium such as soils and plants is required. In addition, the site contamination assessment in the catchment where the abandoned iron mine is situated is actually needed.

In order to identify the potential location of high arsenic contamination in the iron mine surrounding area, the major approach included fuzzy overlay mapping in ArcGIS and surface interpolation of the data derived from field sampling and analysis of arsenic content by spatial anisotropy approach.

Consequently, the primary objectives of this research were (1) to identify the most potential locations of high arsenic contaminant and (2) to verify the most potential locations by surface interpolation of the studied arsenic content in such identified area.

### 1.1. Study Area

**Figure 1:**
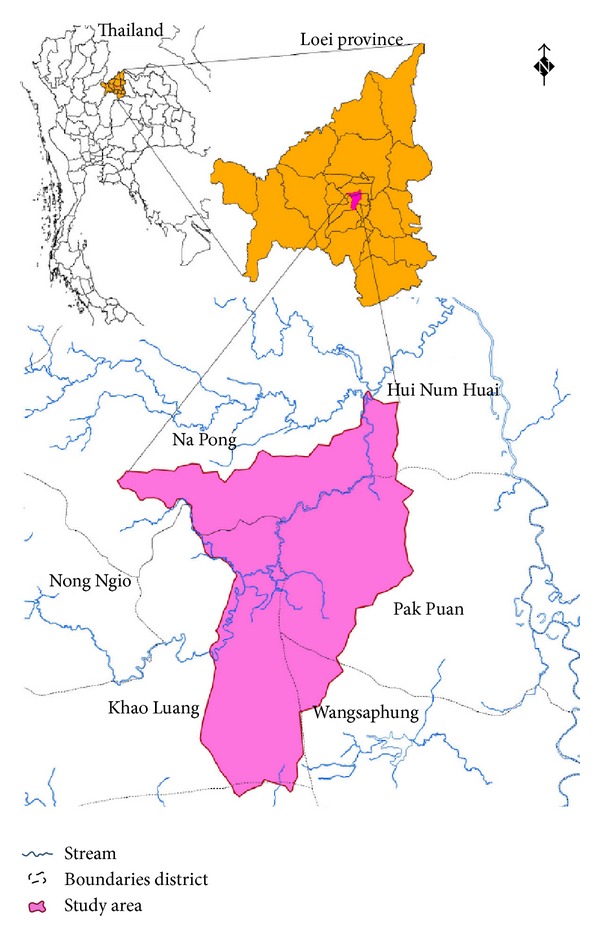
Study area at Wangsaphung district, Loei province, northeast Thailand.

**Figure 2:**
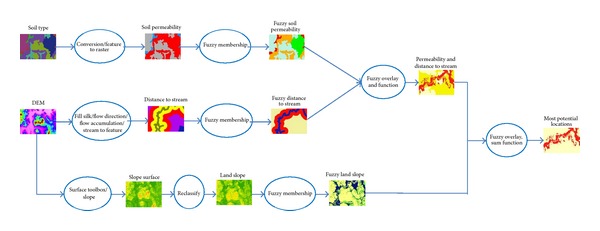
Methodology chart for fuzzy overlay approach.

The study area covered the area surrounding the abandoned iron mine situated at the catchment in Wangsaphung district, Loei province, the northeastern region of Thailand as shown in Figure 1. Most area in this catchment is plateau area with the elevation of about 250–300 meters above mean sea level (mmsl). Iron mine is situated in the east of the catchment at the elevation of 250 mmsl. Within three-kilometer radius of the iron mine, there are four villages, including Ban Na Nong Bong, Ban Huai Phuk, Ban Nam Huai, and Ban Tio Noi, as shown in Figure 2. Based on the iron mine location, Ban Tio Noi is the nearest village and Ban Huai Phuk is the farthest village located 3.5 kilometers in distance at 251 mmsl elevation and located about 4.5 kilometers in distance at 271 mmsl elevation, respectively. Most of land use in the study area is paddy rice field and crop cultivation such as banana, tapioca, nut, and rubber.

Within the study catchment there are many small waterways flowing from the high elevation at the top of plateau, about 500–650 mmsl, flowing to the low elevation area and merging to be one stream, namely, Huai stream, passing through the villages downward the Loei River which then joins the Mekong River.

## 2. Materials and Methods 

The conceptual framework of this study is approached with fuzzy overlay mapping to identify the potential arsenic contamination locations and verification of such potential locations with field sampling and analysis of arsenic content in conjunction with the surface interpolation by spatial anisotropy.

### 2.1. Fuzzy Overlay Approach

The source maps selected for fuzzy overlay approach in ArcGIS are based on the factors determining the most potential locations of arsenic contamination which were as follows.

- Land slope: the raster layer of elevation variability is used for slope steepness classification, which affects the rate of lateral movement.
- Soil type: the percentage of sand in soil significantly determines the rate of percolation of water into the groundwater.
- Distance to stream: the raster layer representing the distance from the main stream of each grid cell in the map is used to examine how far the movement is required for the water body.

Raster data of land slope and distance to stream could be created from DEM (digital elevation model) of a cell size of 5 × 5 m. Both layers are continually spatial factors for determining how much water is contaminated by surface runoff process at a site where the stream reaches. The higher slope with shorter distance to stream and sandy soil (higher permeability) make more opportunities for movement of arsenic through the soil pore by surface runoff and then to the stream or store at the plain area along the stream bank. Raster layers of DEM and soil type could be collected from Land Development Department, Ministry of Agriculture and Cooperatives of Thailand. Maps overlay is illustrated in Figure 2.

### 2.2. Sampling and Analysis of Arsenic Content

**Figure 3:**
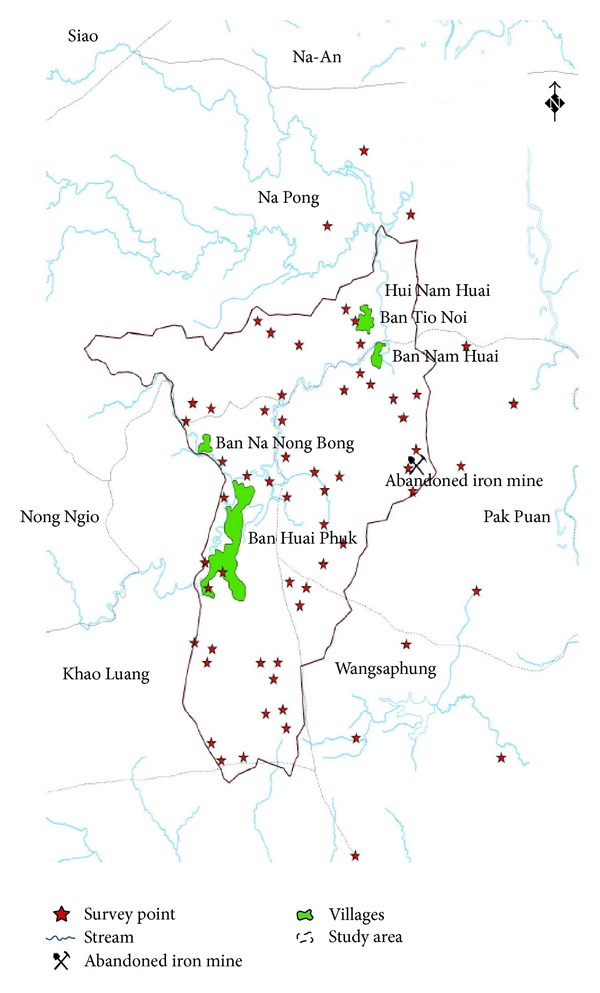
Sampling locations in the study catchment (inside catchment).

Soil samples were taken from the potential area derived from the fuzzy overlay mapping. A total of 51 soil samples collection are illustrated in Figure 3. At each location, soil samples were taken at the depth of 0.00–1.00 m from the surface ground level. Methods of drilling and collecting soil samples were performed in accordance with the guidance of American Society for Testing and Materials [16]. Each soil sample was wrapped with aluminum foil sheet and coated with paraffin to protect against the moisture loss and oxidizing reaction that might occur during carrying to the laboratory for analysis. Analysis for temperature, pH, and oxidation-reduction potential (ORP) was made on site. All soil samples were analyzed for arsenic and iron content, OC, CEC, soil type, and its associated parameters such as moisture content, and unit weight. Arsenic contents were analyzed using Inductively coupled plasma mass spectrometry (ICP-MS) method. This technique provides high precision determination of substance, even metallic or nonmetallic, from the relatively small amount of samples [17, 18]. Soil type was classified using mechanical sieve analysis and hydrometer test. Soil group name associated with soil symbol was designated as recommended by Unified Soil Classification System [19].

### 2.3. Potential Locations Using Fuzzy Overlay

Fuzzy overlay technique employs the principle of fuzzy logic to solve traditional overlay analysis applications in geographic information system (GIS) such as site selection and suitability models. Fuzzy logic is an approach to computing based on “degrees of truth” rather than the usual “true or false” (1 or 0). It is based on the logic of set theory, in which one can traditionally determine whether a value is a member of a set or not. A variation on set theory allows specifying the likelihood that a given value is a member of the set rather than merely specifying whether the value is either in or out of the set [20]. A numeric is used in fuzzy logic with 1 representing full membership in the set and 0 representing nonmembership. Source layer values using fuzzy overlay approach will be assigned corresponding values on this continuous scale between 0 and 1 according to the likelihood that they have membership in the set. These values are known as “fuzzy membership” values [21].

Fuzzy overlay process composes 4 steps: collecting source layers, assigning fuzzy membership values for each layer, combining the fuzzy layers, and evaluating the results. The result is a layer showing the locations most likely and least likely to be contaminated with pollutants. In this research the source layers are land slope, distance to stream, and soil types specifically concentrated on soil permeability. These three layers are continuous data, called raster maps, of which distance to stream and land slope layers were created from DEM of cell size of 5 × 5 m. Figure 2 presents the procedures of how to establish raster map of* distance to stream and land slope* from DEM. To create* a raster map of soil permeability*, the soil type layer was brought as an input raster in the* Conversion tool*/*to Raster*/*Feature to Raster* in toolbox of ArcGIS, the GIS software, also shown in Figure 2.

#### 2.3.1. Assigning Fuzzy Membership

For each source layer, the likelihood that each observed value is a member of the defined set of most potential locations for arsenic contaminant could be specified based on the values for that criterion. Likelihood is indicated by assigning a value on a scale of 1 (very likely) to 0 (not likely). For example, if flat land slope is suitable and steep land slope is not, a value of 1 is assigned to the flat slope and 0 to the steep slope. Between flat and steep slopes, the values are assigned between 1 and 0, accordingly. A new layer, or map, is created corresponding to the fuzzy membership values ranging from 1 to 0. Since there are many observed values in continuous raster layers, it would be more convenient to assign fuzzy membership using mathematical function, which is the relationship between observed values and fuzzy memberships. There are several builtin mathematical functions in ArcGIS such as* linear, small, large, MS small*, and* MS large*.* Small* and* large* fuzzy function in ArcGIS have been used for assigning fuzzy memberships to the observed values or source values when the relationship between observed values and fuzzy membership is not linear. In this context, fuzzy small and fuzzy large function are used to capture nonlinear relationships between observed values and fuzzy membership values. To assign high fuzzy membership values to small observed values, the* small* function will be used. Conversely,* large* function will be used to assign the high fuzzy membership values to large observed values. Following are the assignment details for each source layer in this research.

- Distance to stream: distances to stream were reclassified to be six buffer distances as 100, 500, 1000, 1500, 2000, and 6000 m from the stream and then were assigned for fuzzy memberships with the function* small*. Parameters inputs in the function* small* are 500 for the midpoint, 10 for spread, and* very* for hedge.
- Land slope: land slopes were reclassified into eight groups from low to high degree of slope as* flat to nearly flat, slightly undulating, undulating, rolling, hilly, steep, very steep, and extremely steep,* respectively. Fuzzy memberships were then assigned to those classified layers using the function* small* with the input parameters as 5* in degree of slope* for* midpoint*, 10 for spread, and* very* for hedge.
- Soil permeability: fuzzy memberships were assigned to soil permeability using the function* large*. Parameters were 3.8 mm/hr, 5, and* very* for midpoint, spread, and hedge, respectively.

**Figure 4:**
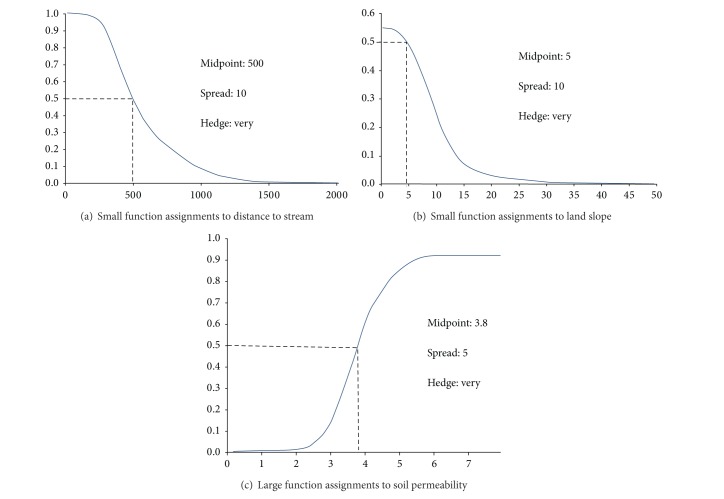
Parameters input for fuzzy small and large for assigning fuzzy memberships.

For the* small* function, the midpoint parameter is the observed value that is assigned for the fuzzy membership value of 0.5, and the spread parameter is the decreasing rate of the fuzzy membership values. The spread defines how tightly the assigned fuzzy membership values cluster around the midpoint. With a large spread value, the fuzzy values decrease rapidly from the midpoint. In case of* the large* function, midpoint parameter represents the observed value that is assigned for the fuzzy membership value of 0.5, the same as the* small* function, but the spread parameter controls the rate at which fuzzy membership increases from low to high. Usually, the spread is defined as a value between 1 and 10 [20].  Figure 4 illustrates the fuzzy function* small* and fuzzy* large* and their parameters used for assigning fuzzy memberships to each source layer.

#### 2.3.2. Combining Fuzzy Membership Layers

The overall likelihood of membership in each of the defined sets can be created by combining all fuzzy membership layers, called fuzzy overlay. Fuzzy operators used for combining membership layers in this research are fuzzy AND and Fuzzy SUM. With the AND operator, the output cell will represent the minimum value from all the input fuzzy membership layers, whereas the output values of SUM operator represent the membership value of a cell in each layer as the results of subtracting fuzzy membership of each observed map from 1, followed by multiplying them and then subtracting from 1.

To accomplish the fuzzy overlay in this study, fuzzy AND was first utilized to combine the fuzzy membership layer of soil permeability and distance to stream, and then that result was combined with fuzzy membership layer of land slope using fuzzy SUM, as the process shown in Figure 3.

For this study, the following values are used subsequently.

- In The distance to stream layer and land slope layer, the* small* function gives the high fuzzy membership values to the small observed values. By applying the input values of 500 as midpoint, 10 as spread, and* very* for hedge, the fuzzy* small* function assigned high fuzzy membership values to the distance to stream that is far from stream less than 500 meters. The decreasing rate of fuzzy membership values is controlled by the “spread.” If we take the spread parameter with high value in fuzzy small, the fuzzy values will steeply decrease when lower than midpoint.
- For land slope layer, the input parameters are of 5 as* Midpoint*, 10 as* Spread* and “*very”* for* Hedge*. Fuzzy small function gave high fuzzy membership values for land slope that are less than 5 degrees which cover slope classes of* flat to nearly flat* and* slightly undulating*. The other types of slope have been assigned rapidly decreased fuzzy values due to large spread input.
- In the soil permeability layer, the fuzzy large function was used because more permeable capacity of soil would get more chances for arsenic transportation and the relationships between permeability values and the fuzzy membership values are not exactly linear. The midpoint of 1.3 exhibits that the fuzzy membership of 0.5 would be assigned to permeability of 1.3 mm/hr and greater value of fuzzy memberships would be assigned for the higher permeability. The spread of 5 controls the rate at which fuzzy membership increases gradually from low to high.
- The input values for midpoint and spread as described above are based on the expert knowledge, followed by iterative process to modify some fuzzy membership values of the source layers to achieve the best results.

**Figure 5:**
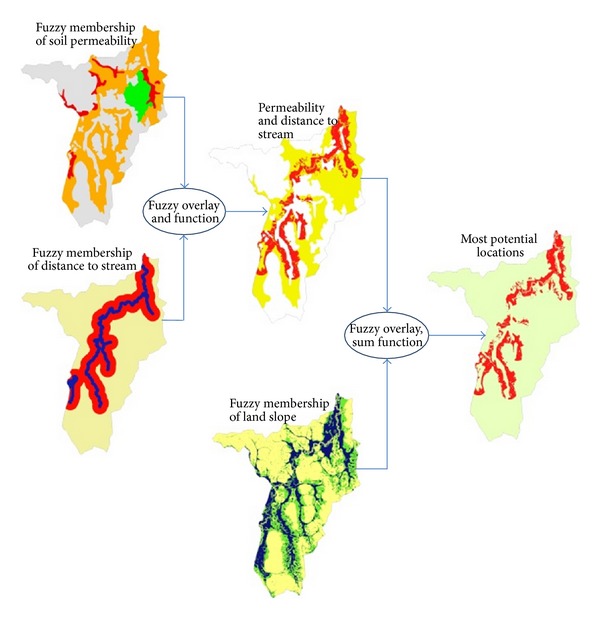
Fuzzy membership layers and process of overlay.

Figure 5 exhibits the layer maps of assigned membership of each data source and next process of their combinations through the use of fuzzy overlay. Fuzzy overlay result of fuzzy membership layers of soil permeability and distance to stream utilizing the AND operator gives the specific areas that are most likely close to stream and associated with the soil condition of high permeability. However, some locations in that result are of high elevation compared to the elevation of the abandoned iron mine which is anticipated as being anthropogenic source of arsenic contaminant. To obtain more reliable result, the fuzzy membership of land slope was overlain with the foregoing result through the fuzzy SUM.

### 2.4. Spatial Anisotropy Assessment

To assess the level of contamination, arsenic concentrations in soils of 51 points in the study area were brought to create surface interpolation using geostatistical techniques, namely, spatial anisotropy. These techniques can not only give a prediction surface but also provide measure of uncertainty or accuracy of the prediction so that they are more reliable than the deterministic technique. In general, the process of establishing surface interpolation is composed of calculating the empirical semivariograms fitting a model and makes a prediction. Kriging, synonymous with “geostatistics” in spatial statistics, is based on the assumption that things that are close to one another and are more alike than those farther away; it generally is called as a spatial autocorrelation. The empirical semivariogram is a mean to explore this relationship. Pairs that are close in distance should have a smaller measurement difference than those farther away from one another [21, 22]. The extent that this assumption is true can be examined in the empirical semivariogram. Semivariogram can be computed for all pairs of locations separated by distance* h*, as follows:

(1) Semivariogram(distance  h)=0.5∗average[(value  at  location  i        −value  at  location  j)2].

There are several models that can be used to fit the empirical semivariogram. Those are, for example, circular, spherical, tetraspherical, exponential, Gaussian, and so forth. Which type is the best model can be determined from the following diagnostics.

- The mean prediction error should be near zero to assure that the prediction errors are unbiased. Also, the standardized prediction errors, that is, the prediction error divided by prediction standard errors, should be near zero.
- The root-mean-square prediction errors should be small. The smaller the root-mean-square the better prediction error.
- The average standard errors should be close to the root-mean-square prediction errors. If the average standard errors are greater than the root-mean-square prediction errors, then there is overestimating in the variability of the predictions; if the average standard errors are less than the root-mean-square prediction errors, then there is underestimating in the variability of the predictions. Another way is to check the root-mean-square is that standardized error should be close to 1.

Crossvalidation chart in ArcGIS can give these diagnostics for testing how well the model predicts the values at unknown locations [22].

For the field data of arsenic concentration and the assumption of exactly unknown but constant mean, the ordinary kriging, one of the geostatistical methods, is chosen to predict the values at the unmeasured points. By examining semivariogram in any directions, it was found that the shape of semivariogram curve varies more with direction, so that the anisotropic approach has been selected as an application in ordinary kriging. In addition, the spherical type of fitting model for semivariogram is appropriate through testing the diagnostics described above.

## 3. Results and Discussion

**Figure 6:**
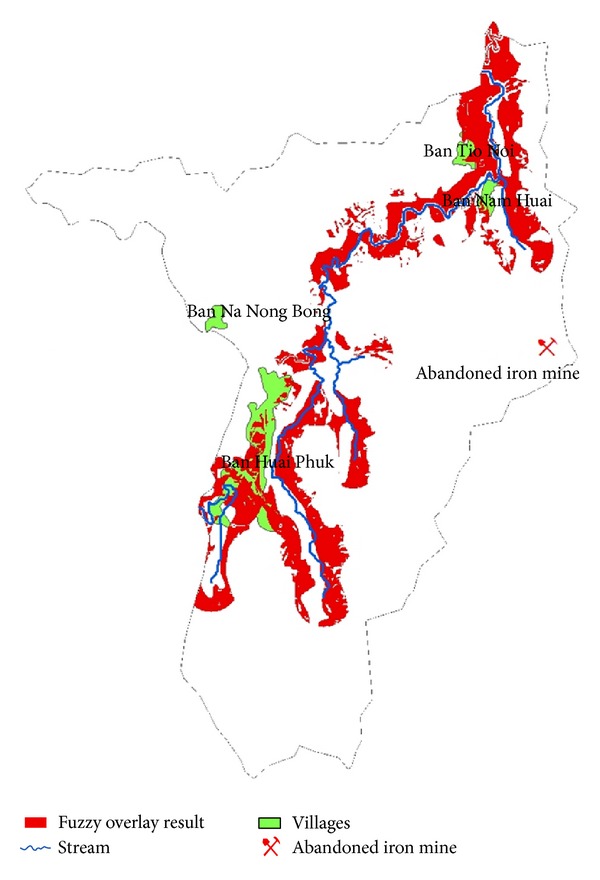
Fuzzy overlay result exhibiting the most potential area of high arsenic content.

Based on fuzzy overlay approach, the results shown in Figure 6 exhibit the potential areas of the most contaminated location with arsenic. Such areas demonstrate more chances of capturing, accumulating, or being the pathway of any polluted materials transported by water because the locations are low in elevation with flat slope, high soil permeability, and distance adjacent to the stream. There are three areas being the most potentially contaminated with the high concentration of arsenic. The first area is adjacent to Ban Huai Phuk, 4.5 kilometers in the west of the iron mine. The second area is at the border of the deciduous forest, 2.8 kilometers in the northwest of the iron mine. The third area is Ban Tio Noi and Ban Nam Huai, 3.5 kilometers in the north of the iron mine. It thus preliminarily indicated Ban Huai Phuk, Ban Tio Noi, and Ban Nam Huai being as threatened by high level of arsenic. In order to ascertain the fuzzy overlay results, this study had investigated arsenic concentration in soil for 51 sapling points within the study catchment (see Figure 3). Surface interpolation by spatial anisotropy approach was employed for verification of the Fuzzy Overlay outputs.

Based on descriptive statistics, arsenic concentration in soils within study catchment was 4.75 ± 4.59 mg/kg as stated in Table 1.

**Figure 7:**
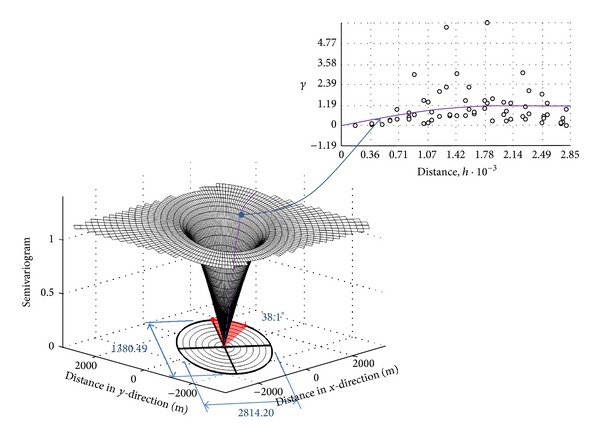
Semivariogram in spatial anisotropy. The top right represents the fitted curve of semivariogram in that direction.

Surface interpolation of arsenic content in soils was produced using ordinary kriging. After detrending and taking the log transformation to arsenic data, empirical semivariogram model was then built from semivariogram scatter plots. Type of spherical anisotropic model was selected as fitted curves for semivariogram models because arsenic data changed not only with the distance but also with the direction. Through the use of MATLAB, three-dimensional anisotropic models of semivariogram could be built, which illustrate semivariogram curves and their parameters in all directions [23]. For example, one semivariogram model of arsenic dataset, shown in Figure 7, presents the values of 1.124 for sill, 1380.49 m for minor range, 2814.20 m for major range, and 38.1° due north for directional angle. Crossvalidation, which helps make an informed decision as to which model provides the best predictions, exhibits the value of mean prediction errors, mean standardized prediction errors, root-mean-square prediction errors, and average standard errors as 0.2485, −0.02989, 3.526, and 3.416, respectively. Consequently, the surface interpolation obtained gives reliable prediction of arsenic content in soils at the unmeasured points.

**Figure 8:**
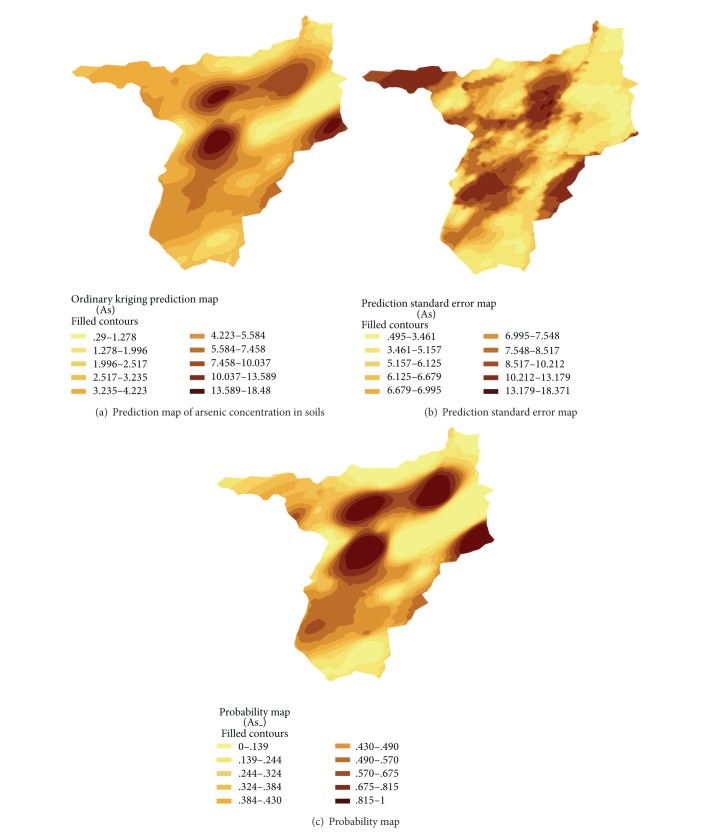
Surface interpolation obtained from spatial anisotropy using ordinary kriging.

**Figure 9:**
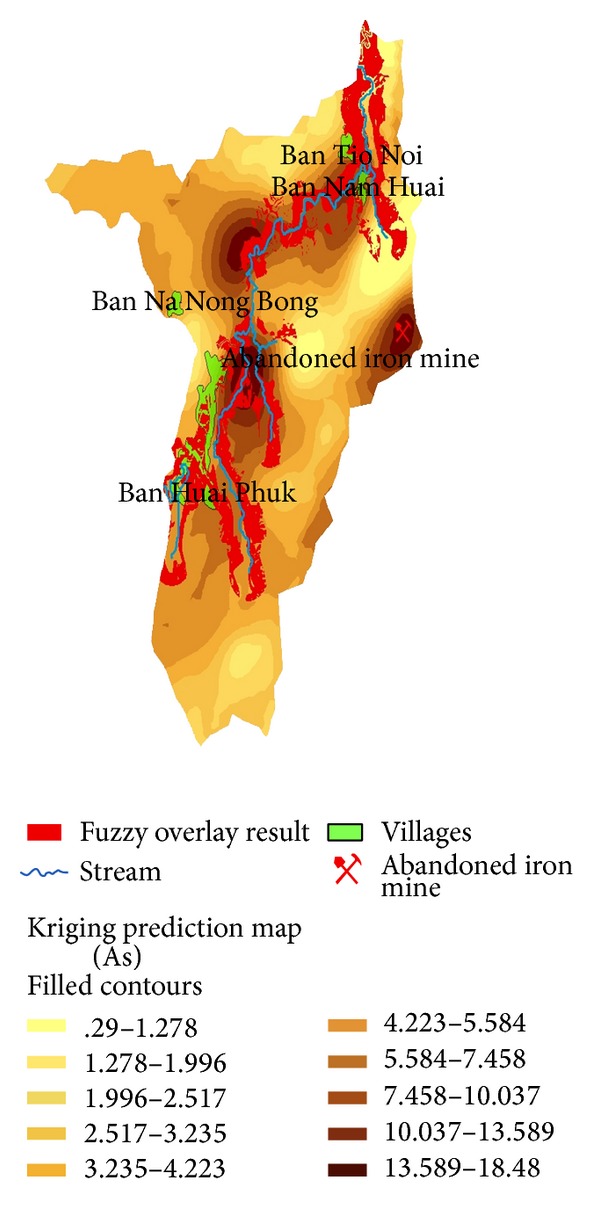
Result of fuzzy overlay match on surface interpolation.

Figure 8 illustrates the results of surface interpolation using ordinary kriging as previously described. It contains 3 maps including prediction map, prediction standard error map, and probability map. The prediction map, in Figure 8(a), presents four local zones of high arsenic content. Placing the prediction map on the fuzzy overlay result, as illustrated in Figure 9, it is obviously seen that, except the zone of iron mine, the other three zones of high arsenic content match the areas in which they were found by the fuzzy overlay approach. Evidently, the fuzzy overlay approach can prove being the most effective way for providing preliminary information necessary for further field works.

In case of assessment, only prediction model might not be adequate to indicate the contaminant area precisely, and a decision about the classification of safe and unsafe areas with predictions map alone could be inaccurate. To assure the prediction results, prediction standard error and probability maps have been taken into account in considering highly contaminated areas. Prediction standard error in dark-colored areas as shown in Figure 8(b) at bottom right is large, indicating much larger variability in prediction. In this case, the usefulness of probability map in Figure 8(c) helps assure the most contaminated areas obtained from the prediction map. The probability map presents the probability of each area that arsenic could exceed the threshold value of 3.9 mg/kg, MCL for agricultural use.

As illustrated in the prediction map, in Figure 8(a), Ban Tio Noi, Ban Nam Huai, and northeast area of Ban Huai Phuk are in the areas of high arsenic content. Also, the probability map, in Figure 8(c), presents 81.5–100%, ensuring the most contaminated arsenic area is at those three villages. Arsenic content in soils at such areas generally exceeded 3.9 mg/kg, the MCL for agricultural use. Within the buffer zone of 500 m from each village, quantity of arsenic in soils and plants is grouped as presented in Table 2. Ban Nam Huai seems to expose more serious risk than other villages because of higher mean value and smaller standard deviation. In paddy rice field, both soil and rice in these three areas should be further intensively investigated as rice is the pathway media to human consumption.

## 4. Conclusion

Fuzzy overlay is an effective method to preliminarily identify the most potential areas contaminated with arsenic. With the data sources of the distance to stream, land slope, and soil permeability, fuzzy overlay, which is the application in ArcGIS, could give the contaminated locations. In this context fuzzy function and the functions* small* and* large* were used to assign fuzzy membership and then we used fuzzy* AND* and fuzzy* SUM* to combine layers of fuzzy membership. The function* Small* was applied for the distance to stream and land slope as the low source values were the most suitable. The function* large* was applied for soil permeability because the large source values were the most suitable. Result of the fuzzy overlay displayed three locations of high potential arsenic contamination. Accordingly, those locations were in conformance with the area of high arsenic content derived by the spatial anisotropy assessment.

The fuzzy overlay has thus been proved to be straightforward approach for finding the preliminary site study. In general, this method could be applied for any heavy metals or any polluted materials taken with water passing through soil media.

Spatial anisotropy assessment exhibited four zones of high arsenic content in soils. Regardless of iron mine zone which can play major role as an anthropogenic source, the other three zones cover three villages including Ban Huai Phuk, Ban Nam Huai, Ban Tio Noi. Ban Nam Huai is the most risky area because of its high mean value of arsenic contaminant and narrow range of standard deviation. Arsenic content in soils at Ban Nam Huai is 7.19 ± 2.86, exceeding the MCL of 3.9 mg/kg for agricultural soils. Ban Huai Phuk and Ban Tio Noi have also high values and exceeding MCL, but are lower than Ban Nam Huai. The line government agencies should place effective preventive measures and appropriate remediation at this high contaminated area, particularly those three mentioned villages.

## Figures and Tables

**Table 1:** Quantity of arsenic concentration in soils at the inside and outside of the study catchment, other parameters, and their basic statistics.

	Depth (m)	Measured range (mean value ± SD)	Median	Skewness	Kurtosis
As (mg/kg)	0.00	0.29–18.48 (4.75 ± 4.59), *N* = 51	3.41	1.67	1.93
Fe (mg/kg)	0.00	2800–38500 (16300 ± 8396), *N* = 51	14500	0.42	−0.53
pH	0.00	4.20–8.53 (7.44 ± 0.80), *N* = 51	7.67	−2.48	4.27
Temp (°C)	0.00	23.90–39.50 (29.70 ± 3.62), *N* = 51	29.10	0.96	0.69
ORP (mV)	0.00	−104.80–413.70 (225.78 ± 106.85), *N* = 51	248.80	−1.14	1.97

**Table 2:** Arsenic content in soils within buffer zone of 500 meters from each village (mg/kg).

Villages	Measured range (mean value ± SD)	Plant
Huai Phuk	4.05–12.04 (6.60 ± 3.04)	Rice paddy, corn, longan, mix orchard, mix field crop

Na Nong Bong	2.11–4.64 (3.62 ± 0.80)	Rice paddy, mix field crop, dense deciduous forest

Nam Huai	2.93–10.79 (7.19 ± 2.86)	Rice paddy, corn, mix orchard

Tio Noi	1.95–9.23 (4.90 ± 2.67)	Rice paddy, mix orchard
